# Evolution of de Winter syndrome to Wellens syndrome: a case report and literature review

**DOI:** 10.3389/fcvm.2024.1415306

**Published:** 2025-01-09

**Authors:** Fei Wang, Xuesong Zhang, Huihui Pang, Yuehai Wang

**Affiliations:** ^1^ Department of Cardiology, Shandong Corps Hospital of Chinese People’s Armed Police Forces, Jinan, Shandong, China; ^2^ Cardiology Department and Experimental Animal Center, Liaocheng People’s Hospital of Shandong University and Liaocheng Hospital Affiliated to Shandong First Medical University, Liaocheng, Shandong, China; ^3^ School of Clinical Medicine, Shandong Second Medical University, Weifang, Shandong, China

**Keywords:** de Winter syndrome, Wellens syndrome, proximal LAD lesion, culprit lesion, specificity, incomplete occlusion, short-term

## Abstract

Both de Winter syndrome and Wellens syndrome mainly indicate severe stenosis in the proximal segment of the anterior descending coronary artery. However, as research deepens, the accuracy and specificity of diagnosing proximal left anterior descending coronary artery (LAD) culprit lesions separately by de Winter syndrome or Wellens syndrome are challenged. The patient in this case developed both syndromes in a short period of time, and imaging showed significant stenosis of the proximal LAD, indicating a culprit lesion. The successive appearance of these two special electrocardiogram changes may increase the accuracy and specificity of diagnosing LAD as a culprit lesion, and the short-term occurrence of these two special electrocardiogram changes also suggests that the culprit lesion may be incomplete occlusion. In addition, de Winter syndrome is prone to missed diagnosis, while Wellens syndrome is prone to misdiagnosis or underestimation of its risk.

## Introduction

De Winter syndrome is a special type of acute myocardial infarction that indicates severe lesions in the proximal segment of the left anterior descending coronary artery (LAD). Early identification of it is of great significance for preventing sudden death from coronary artery disease (CAD). However, de Winter syndrome is an easily overlooked and missed diagnosis. Wellens syndrome is prone to progress to acute ST segment elevation extensive anterior myocardial infarction. These two special types of electrocardiogram (ECG) changes mainly indicate severe stenosis in proximal LAD and are considered to be critical conditions, such as ST segment elevation myocardial infarction (STEMI) (1–4). In clinical practice, de Winter or Wellens syndrome is usually detected separately, and there are few reports of both types of ECG patterns occurring consecutively in the same patient. However, our hospital recently treated a patient with non-ST segment elevation myocardial infarction (NSTEMI) and recorded in detail this rare example in the patient's ECGs before and after the onset of the disease. Moreover, by reviewing literature and analyzing its mechanism, we further deepened our understanding of the clinical significance of these two ECG manifestations.

## Case presentation

**Figure 1:**
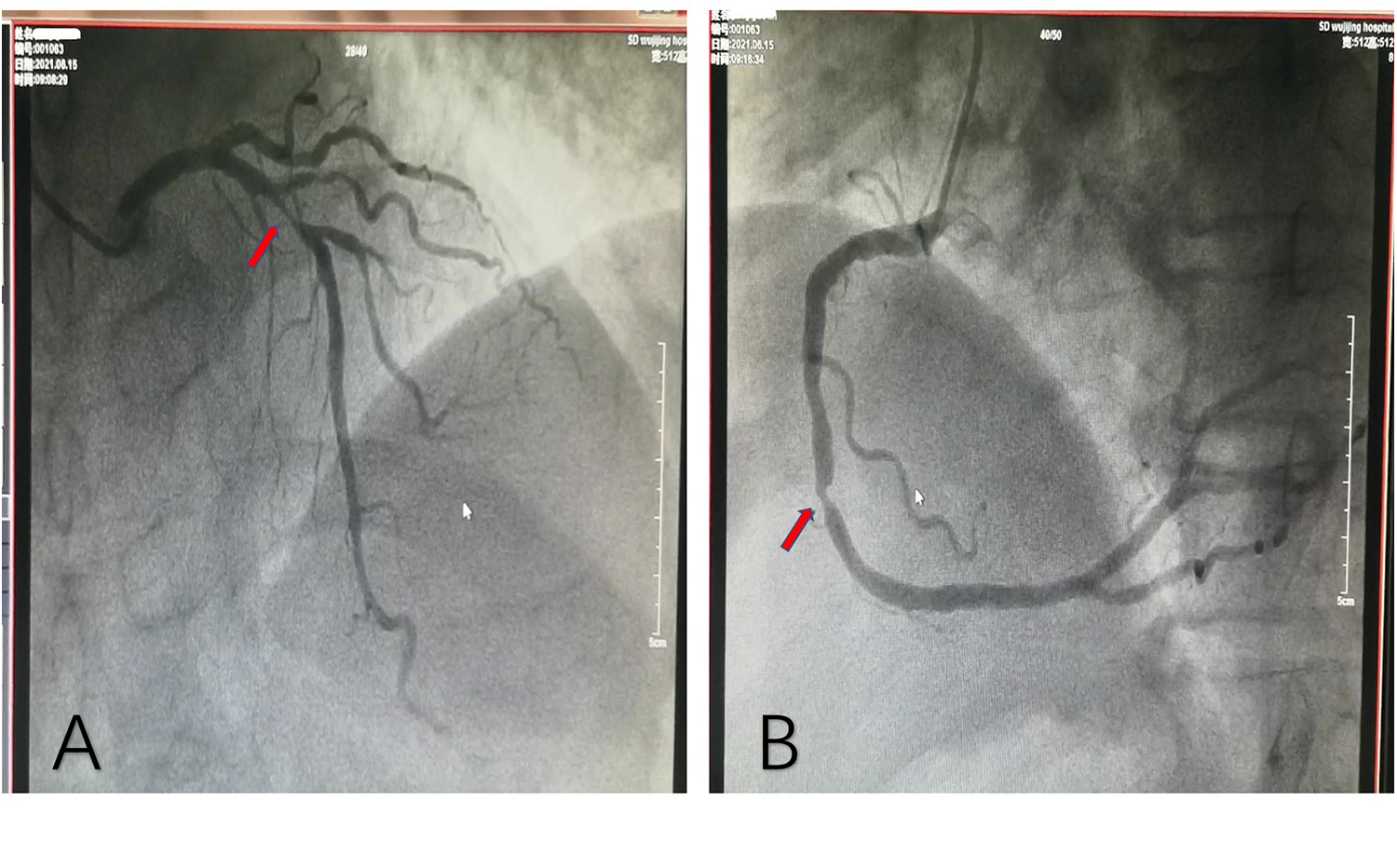
Coronary angiography. **(A)** Stenosis of 95% in the proximal LAD, with blurred lesion margins. **(B)** Stenosis of 75% in the middle RCA, with a regular lesion margin. LAD, left anterior descending artery; RCA, right coronary artery.

**Figure 2:**
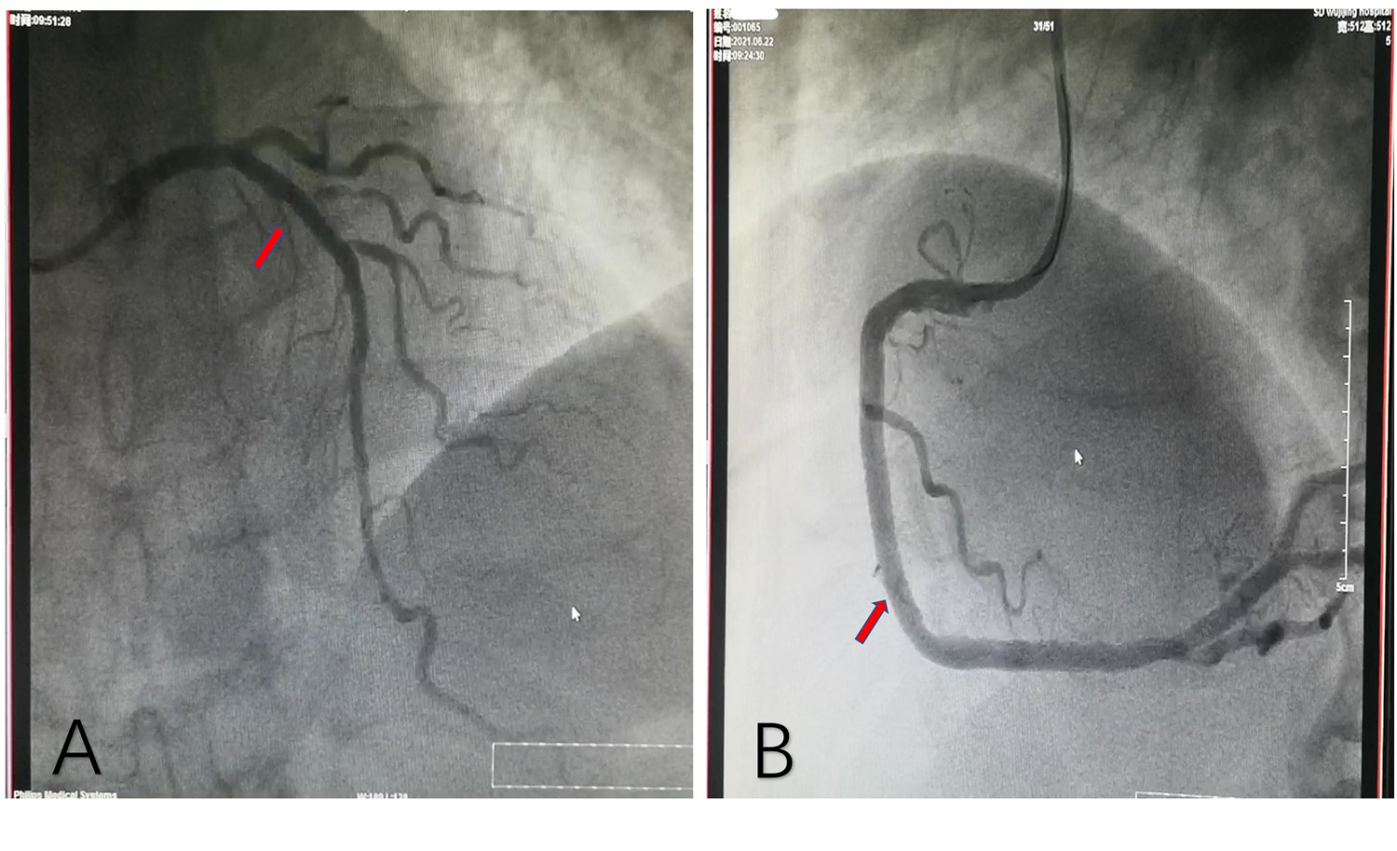
Coronary angiography after stent implantation. **(A)** One stent implanted in the proximal LAD. **(B)** Another stent implanted in the middle RCA. LAD, left anterior descending artery; RCA, right coronary artery.

The patient, a 77-year-old man, was admitted due to “sudden unstable chest pain.” Resting for 15–20 min could alleviate the patient's chest tightness in the 2 months before admission. Half an hour before admission, the patient experienced chest tightness again during physical activity and was admitted to the emergency department with chest tightness lasting for 40 min. This patient had a history of hypertension for more than 10 years and oral administration of 80 mg valsartan and 5 mg amlodipine tablets daily has controlled his blood pressure (BP) to normal. The patient has a smoking history of 50 years, accompanied by 10 cigarettes per day, and his mother has a history of cerebral hemorrhage. Upon admission, physical examination showed a BP of 154/75 mmHg and heart rate of 68 beats/min; no significant abnormalities were observed during cardiopulmonary auscultation. There was no edema in both lower limbs. An ECG examination immediately upon admission showed sinus rhythm and ST-T changes, and the patient was preliminarily diagnosed with acute coronary syndrome (ACS). The patient refused a coronary intervention examination and received dual antiplatelet, statin, and nitrate therapy. The blood TNI titer was monitored and increased to 0.727 ng/ml (0–0.023 ng/ml) 12 h after admission. Fasting blood glucose was 4.95 mmol/L. The titer of low-density lipoprotein cholesterol was 2.54 mmol/L. The blood indicators of liver and kidney function were found to be normal. Cardiac Doppler ultrasound indicated normal cardiac structure and function. Three days after admission, the patient agreed to undergo coronary intervention treatment. Coronary angiography (CAG) showed 50% stenosis in the distal left main trunk (LM), 95% stenosis alongside a blurred lesion edge in the proximal LAD (Figure 1A), and approximately 75% stenosis in the middle segment of the right coronary artery (RCA) (Figure 1B). LAD was identified as the criminal vessel and one stent was inserted (Figure 2A). Later, one stent was inserted into the middle RCA (Figure 2B). After discharge, the patient underwent standardized secondary prevention and treatment for CAD and no further chest tightness occurred.

**Figure 3:**
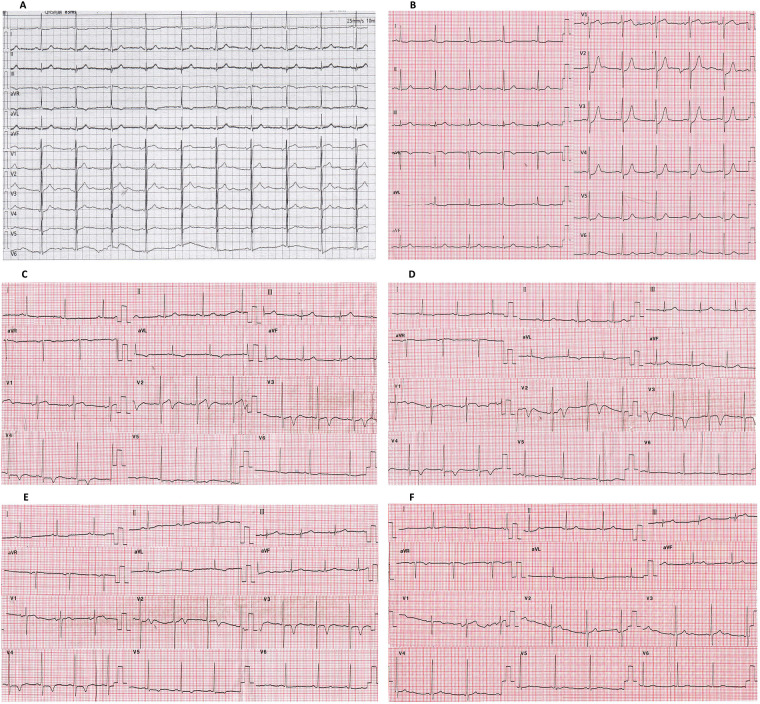
Changes in the ECG of this patient. **(A)** Patient's asymptomatic ECG before hospitalization. **(B)** ECG at admission (persistent chest tightness for 30 min). **(C)** ECG after 1 h of hospitalization (chest tightness relieved for 20 min). **(D)** ECG after 12 h of hospitalization. **(E)** ECG after 4 days of hospitalization (1 day after percutaneous intervention). **(F)** ECG 1 month after percutaneous intervention. De Winter syndrome is shown in **(B)**. Wellens syndrome is shown in **(C**–**E)**. ECG, electrocardiogram; PCI, percutaneous intervention.

The changes in the ECG pattern of this patient before and after hospitalization were identified. Compared with the ECG (Figure 3A) 3 months before admission, the ECG of the patient at admission (continuous chest tightness for 30 min) showed an upward sloping depression of 2 mm in ST_V2–4_ (J-point depression) accompanied by symmetrical tall and peaked T-waves and an elevation of nearly 1 mm in ST_AVR_ (de Winter syndrome) (Figure 3B). The patient's chest tightness lasted for 40 min, and the ECG pattern 30 min after admission (chest tightness relieved for 20 min) showed that the changes in the ST segment disappeared, replaced by bidirectional T_V2_ and inverted T_V3−V4_ (Figure 3C). The T-wave changes persisted for more than 4 days (Wellens syndrome) (Figures 3C–E). The patient’s ECG pattern showed an upright T_V2–4_ (Figure 3F) 33 days after the onset of the disease.

## Discussion

### Electrocardiogram changes in de Winter syndrome and Wellens syndrome

De Winter syndrome and Wellens syndrome are two distinct ECG patterns named after their respective discoverers. The main feature of de Winter syndrome is an upward sloping ST_V1−V6_ depression (1–3 mm J-point downward shift), accompanied by symmetrical tall and peaked T-waves. Most cases also exhibit a ST_AVR_ elevation of 1–2 mm (1, 5). In contrast, the main features of Wellens syndrome are bidirectional or symmetrical and deeply inverted T_V2−V3_ after angina relief, which may extend to leads V4–V6 (5, 6). In this case, the patient's ECG initially showed changes in ST_V2−V4, AVR_ consistent with de Winter syndrome, 30 min after symptom onset. However, subsequent ECGs recorded 20 min to 4 days after symptom relief displayed T_V2−V4_ changes consistent with Wellens syndrome. The patient presented with these two special ECG manifestations in a short period of time.

### Sensitivity and specificity of de Winter syndrome and Wellens syndrome in diagnosing underlying lesions in proximal LAD

Although de Winter syndrome and Wellens syndrome are classified as non-ST segment elevation acute coronary syndromes, both indicate severe stenosis in the proximal LAD and are considered equivalent to STEMI (7, 8). CAG should be performed as early as possible and further revascularization therapy such as percutaneous coronary intervention (PCI) should be performed (6–9).

De Winter syndrome is found in 2% of patients with acute proximal LAD occlusion (1) and 3.4% of patients with acute anterior myocardial infarction (10); however, there are also reports that de Winter syndrome is occasionally found in patients with proximal-RCA occlusion (11), LAD branch embolism (12), LM or left circumflex artery lesions (13), STEMI thrombolysis with LAD occlusion (14), and myocarditis (15). Early studies have shown that de Winter syndrome can predict 95%–100% acute proximal LAD occlusion (16, 17). However, a subsequent meta-analysis showed that de Winter's positive predictive rate for LAD occlusion was only 50%–85.7% (17). Previous studies have shown that Wellens syndrome occurs in 14%–18% of ACS patients (6, 18) and 8.8% of NSTEMI patients (4). Two-thirds of the culprit lesions in Wellens syndrome are located in the LAD and one-third of the culprit lesions in Wellens syndrome are located in the proximal LAD. The sensitivity and specificity for predicting LAD underlying lesions were 24.6% and 96.2%, respectively (4). However, other studies have shown that 31% of patients with Wellens syndrome have normal coronary arteries or experience non-obstructive CAD (3). Therefore, as people's understanding gradually increases, the specificity for diagnosing proximal LAD occlusive lesions, whether it is de Winter syndrome or Wellens syndrome, is being challenged.

There are few reports of consecutive occurrence of de Winter syndrome and Wellens syndrome in the same patient; however, the culprit lesions in the cases reported so far have been located in the proximal LAD and were not completely occluded (19–23). Therefore, we speculate that the sequential presence of these two ECG patterns may not only enhance the accuracy and specificity of diagnosing proximal LAD lesions, but also indicate that the culprit lesion is not completely occluded. Whether this represents a completely occluded lesion that reopened within a short period remains unclear and should be clarified.

### Easily misdiagnosed de Winter syndrome and preferably misdiagnosed or underestimated Wellens syndrome

De Winter syndrome is an electrocardiogram pattern similar to the hyperacute phase of myocardial infarction. Although it holds significant clinical value, the time window for observing this special ECG change is very short, with an average reported time of 1.5 h, as reported in the literature (1). Monitoring the ECG changes of the patient in this case revealed that de Winter syndrome was recorded 30 min after the onset of chest tightness. Wellens syndrome was subsequently recorded 30 min later (after 20 min of chest tightness relief), with an interval of only 30 min between the two different ECG patterns. Wellens syndrome typically occurs after the resolution of angina rather than during symptom onset, and this ECG pattern can persist for several weeks (8). Furthermore, Wellens syndrome is characterized solely by T-wave changes. Without comparison to pre-onset or normal ECGs, these changes can easily be misinterpreted as other conditions or non-specific T-wave changes. In addition, Wellens syndrome is only characterized by T-wave changes, which, in the absence of pre-onset or baseline ECG, can be misinterpreted considered as other conditions or non-specific T-wave changes. In this case, although the patient showed Wellens syndrome on multiple follow-up ECGs after admission, there was no dynamic evolution and the patient remained in an asymptomatic state. Therefore, in clinical practice, de Winter syndrome is often misdiagnosed and Wellens syndrome is easily misdiagnosed or underestimated. This case highlights the importance of vigilance among clinicians when encountering Wellens syndrome on a patient’s ECG. Even if the patient is asymptomatic and there are no dynamic changes in the follow-up ECGs, it is still necessary to investigate recent symptoms of chest tightness or chest pain and any previous ECG changes to identify high-risk patients in a timely manner. The uniqueness of this case lies in the sequential occurrence of de Winter syndrome and Wellens syndrome within a short period of time. These dynamic ECG changes provide sufficient basis for judging the patient's condition.

### Underlying mechanisms of de Winter syndrome and Wellens syndrome

The pathophysiological mechanisms of de Winter syndrome need to be clarified. It is generally believed that the ST changes are hyperacute ECG changes caused by acute total occlusion of the proximal LAD; however, ST elevation does not occur due to short-term reperfusion of coronary artery occlusion or the presence of collateral circulation supply (7, 8). Another study (11) suggests several potential mechanisms for de Winter syndrome, including anatomical variations in Purkinje fibers and delayed endocardial conduction. Ischemic ATP depletion may also play a role, as it prevents the activation of ATP-sensitive potassium channels in the muscle membrane. In addition, the area of transmural ischemia is so large that it fails to generate damaging currents toward the precordial leads and instead produces upward currents to the AVR lead. The mechanism of Wellens syndrome is also not yet fully understood. Potential mechanisms include severe stenosis of the proximal LAD, coronary artery spasm (24), or myocardial reperfusion injury (25), which may cause myocardial stunning or edema after myocardial ischemia (7, 26), resulting in abnormal T-wave repolarization.

The patient in this case presented with sequential ECG changes consistent with de Winter syndrome and Wellens syndrome. The underlying mechanism may involve the formation of blood clots at unstable plaques in the proximal LAD, leading to acute exacerbation or complete occlusion of the lumen and resulting in acute, severe myocardial ischemia. This resulted in de Winter syndrome being displayed on the ECG. The rapid reduction in thrombus size results in decreased vascular stenosis or the rapid reopening of completely occluded vascular lumens. After partial recovery from myocardial ischemia, myocardial stunning occurs, causing abnormal repolarization of myocardial cells and ECG changes, which evolves into Wellens syndrome. After PCI, myocardial perfusion was restored effectively, leading to a gradual improvement in myocardial cell activity normalization of cardiac cell repolarization. As a result, the ECG returned to its pre-hospitalization state. This case demonstrated a progression of ECG changes from “roughly normal → de Winter syndrome → Wellens syndrome → roughly normal,” further deepening our understanding of the clinical significance and pathogenesis of de Winter syndrome and Wellens syndrome.

## Conclusion

The sequential appearance of de Winter syndrome and Wellens syndrome within a short time frame may enhance the specificity and accuracy of diagnosing underlying lesions in the proximal LAD. In addition, it suggests that the LAD may be in a state of non-complete occlusion. De Winter syndrome susceptible to missed diagnosis, while Wellens syndrome is prone to misdiagnosis or underestimation of the patient's condition.

## Data Availability

The original contributions presented in the study are included in the article/Supplementary Material, further inquiries can be directed to the corresponding author.
